# Study of the Photodynamic Activity of N-Doped TiO_2_ Nanoparticles Conjugated with Aluminum Phthalocyanine

**DOI:** 10.3390/nano7100338

**Published:** 2017-10-20

**Authors:** Xiaobo Pan, Xinyue Liang, Longfang Yao, Xinyi Wang, Yueyue Jing, Jiong Ma, Yiyan Fei, Li Chen, Lan Mi

**Affiliations:** 1 Department of Optical Science and Engineering, Shanghai Engineering Research Center of Ultra-Precision Optical Manufacturing, Green Photoelectron Platform, Fudan University, 220 Handan Road, Shanghai 200433, China; 11110720002@fudan.edu.cn (X.P.); 14307130398@fudan.edu.cn (X.L.); 17110720023@fudan.edu.cn (L.Y.); 16210720013@fudan.edu.cn (X.W.); yyjing16@fudan.edu.cn (Y.J.); jiongma@fudan.edu.cn (J.M.); fyy@fudan.edu.cn (Y.F.); 2 School of Arts and Sciences, MCPHS University, 179 Longwood Ave, Boston, MA 02115, USA; lichenphy@gmail.com

**Keywords:** titanium dioxide, phthalocyanine, reactive oxygen species, photodynamic therapy

## Abstract

TiO_2_ nanoparticles modified with phthalocyanines (Pc) have been proven to be a potential photosensitizer in the application of photodynamic therapy (PDT). However, the generation of reactive oxygen species (ROS) by TiO_2_ nanoparticles modified with Pc has not been demonstrated clearly. In this study, nitrogen-doped TiO_2_ conjugated with Pc (N-TiO_2_-Pc) were studied by means of monitoring the generation of ROS. The absorbance and photokilling effect on HeLa cells upon visible light of different regions were also studied and compared with non-doped TiO_2_-Pc and Pc. Both N-TiO_2_-Pc and TiO_2_-Pc can be activated by visible light and exhibited much higher photokilling effect on HeLa cells than Pc. In addition, nitrogen-doping can greatly enhance the formation of ^1^O_2_ and •O_2_^−^, while it suppresses the generation of OH•. This resulted in significant photodynamic activity. Therefore, N-TiO_2_-Pc can be an excellent candidate for a photosensitizer in PDT with wide-spectrum visible irradiation.

## 1. Introduction

Titanium dioxide (TiO_2_) nanoparticles have been widely studied in many fields such as solar cells, electrochromic devices, environment, and biomedicine [1,2]. Recently, researchers have focused on the application of photodynamic therapy (PDT) due to its low toxicity, high stability, excellent biocompatibility, and unique photocatalytic properties. When TiO_2_ is photoexcited upon UV irradiation, hole-electron pairs are generated, which result in the formation of reactive oxygen species (ROS) via the redox reactions of oxygen or water molecules at the TiO_2_ surface. The generated ROS can induce a remarkable photokilling effect against cancer cells [3,4,5]. Furthermore, when doped or modified with different methods, TiO_2_ nanoparticles may become an attractive photosensitizer (PS) under visible light irradiation. In particular, TiO_2_ nanoparticles modified with phthalocyanine have been proven to be promising as PSs with enhanced absorption in the visible region [6,7].

Phthalocyanine and its derivatives, as a second generation of PSs, are known to generate singlet oxygen (^1^O_2_) via energy transfer [8]. Also, there have been some studies showing that TiO_2_ nanoparticles can generate specific ROS such as hydroxyl radicals (OH•) [9] and superoxide anion radicals (•O_2_^−^) [10]. However, little work has been conducted to investigate the generation of ROS by TiO_2_ nanoparticles modified with Pc.

In our previous work [11], nitrogen-doped TiO_2_ nanoparticles (N-TiO_2_) conjugated with aluminum phthalocyanine (Pc) were synthesized by a two-step surface modification method, and this novel material, N-TiO_2_-Pc, exhibited significant photokilling efficiency on cancer cells. The photodynamic activity of N-TiO_2_-Pc is the primary driving force underlying the PDT application, so it is important to demonstrate the photo-induced active species clearly. In this study, the photodynamic activity of N-TiO_2_-Pc was studied by monitoring the generation of ROS and evaluating the photokilling effect upon light in different regions. These results are compared with Pc and non-doped TiO_2_-Pc to reveal the roles of nitrogen-doping and Pc.

## 2. Results

### 2.1. Absorption Spectrum

**Figure 1:**
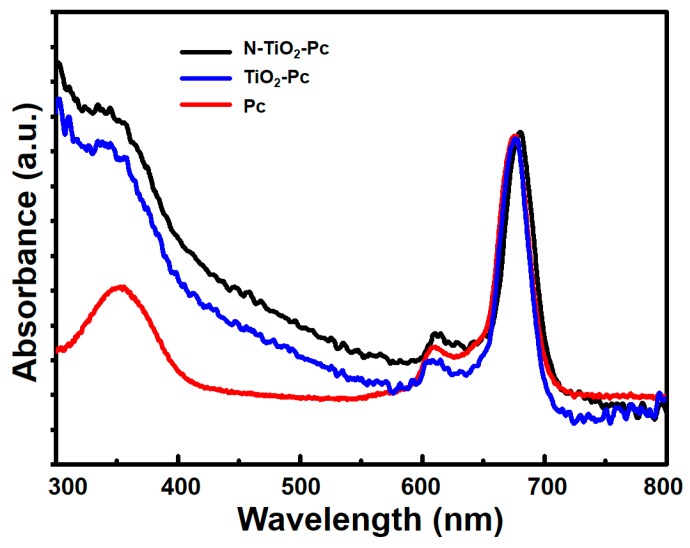
Absorption spectra of N-TiO_2_-Pc (black), TiO_2_-Pc (blue), and Pc (red) in aqueous solutions.

The absorption spectra of N-TiO_2_-Pc, TiO_2_-Pc, and Pc in aqueous solutions are shown in Figure 1. The concentration of Pc in all three samples is the same, which is associated with the similar absorbance around 670 nm of all the samples. Meanwhile, the conjugates of N-TiO_2_-Pc and TiO_2_-Pc both demonstrate higher absorbance in the region of 400–500 nm compared with Pc. It is well known that pure anatase TiO_2_ can only absorb UV light with a wavelength shorter than 387 nm [12]. When TiO_2_ nanoparticles were modified with the amino silanization method, the absorbance in the visible region could be enhanced [13], especially in the blue and green regions [14]. N-TiO_2_-Pc and TiO_2_-Pc were both synthesized based on the amino silanization of TiO_2_ nanoparticles, which leads to enhanced absorbance in the region of 400–500 nm. In addition, N-TiO_2_-Pc shows higher visible absorbance than TiO_2_-Pc due to nitrogen doping, which is in agreement with our previous report [15]. The higher absorption in the visible light region may induce a greater production of ROS and thus a higher photokilling effect on cancer cells.

### 2.2. Production of ROS

**Figure 2:**
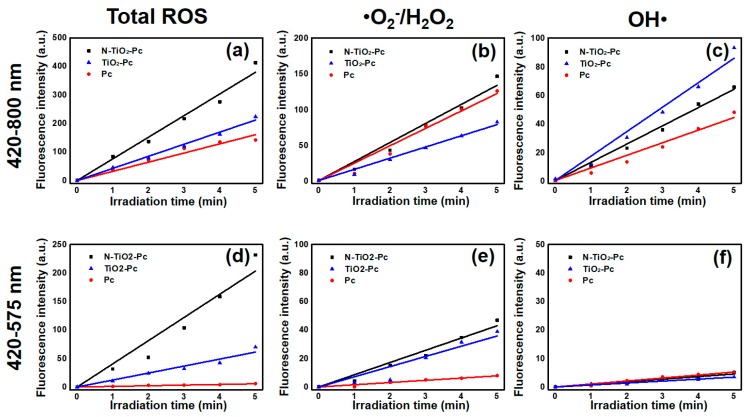
Comparison of photo-induced reactive oxygen species (ROS) by N-TiO_2_-Pc, TiO_2_-Pc, and Pc in aqueous solutions under light irradiation of (**a**–**c**) 420–800 nm (17.8 mW⋅cm^−2^) and (**d**–**f**) 420–575 nm (8.4 mW⋅cm^−2^), where the concentration of Pc is the same in all three samples. Fluorescence intensities indicate the production of (**a**,**d**) total ROS, (**b**,**e**) •O_2_^−^/H_2_O_2_, and (**c**,**f**) OH• as a function of irradiation time.

The ROS generated by N-TiO_2_-Pc, TiO_2_-Pc, and Pc in aqueous suspensions under visible light irradiation were monitored by different ROS-sensitive fluorescence probes. The fluorescence intensities indicated the production of total ROS, •O_2_^−^/H_2_O_2_, and OH•, respectively. The production of ROS increased as a function of light exposure time ranging from 0 to 5 min (Figure 2). For comparison, the concentration of Pc is the same in all three samples.

Under 420–800 nm irradiation, the total ROS production by N-TiO_2_-Pc was higher than those of TiO_2_-Pc and Pc (Figure 2a). The total ROS production of N-TiO_2_-Pc was about 1.8 times than of TiO_2_-Pc and about 2.4 times that of Pc, which agrees well with the visible light absorbance result. N-TiO_2_-Pc induced more •O_2_^−^/H_2_O_2_, while TiO_2_-Pc generated less •O_2_^−^/H_2_O_2_ than Pc (Figure 2b). As shown in Figure 2c, TiO_2_-Pc generated more OH•, was about twice of that of Pc, while N-TiO_2_-Pc produced less OH• than TiO_2_-Pc.

To further study the effect of 420–575 nm irradiation, a 575 nm-shortpass filter was added. It was determined that the power density of the lamp in the range of 420–800 nm was 17.8 mW⋅cm^−2^, and that in the range of 420–575 nm was 8.4 mW⋅cm^−2^, about half of 420–800 nm. Under 420–575 nm irradiation, Pc barely produced detectable ROS. This result indicates that Pc has no absorption in the range of 420–575 nm. Compared with TiO_2_-Pc, the total ROS production of N-TiO_2_-Pc was much higher, around 3.4 times that of TiO_2_-Pc (Figure 2d). This indicates that the photoactivity of N-TiO_2_-Pc is more efficient under this range of visible light. The •O_2_^−^/H_2_O_2_ productions by N-TiO_2_-Pc and TiO_2_-Pc were similar, as shown in Figure 2e. Among the various reactive species, it seems the generation of OH• was not favored (Figure 2f). The reported ROS generated by TiO_2_ included •O_2_^−^, H_2_O_2_, OH•, and ^1^O_2_ [10]. From Figure 2d–f, it can be seen that neither •O_2_^−^, H_2_O_2_, or OH• produced by N-TiO_2_-Pc represent the main contribution of the total ROS upon 420–575 nm irradiation. So, it can be assumed that ^1^O_2_ may be the major composition of the various reactive species.

To further study the contribution of different specific ROS generated by the samples, superoxide dismutase (SOD) and glycerol were used as •O_2_^−^ and ^1^O_2_/•O_2_^−^ scavengers [16,17]. In the presence of specific ROS scavengers, the amount of eliminated •O_2_^−^ and ^1^O_2_/•O_2_^−^ were monitored by the intensity decrease of the fluorescent probe. Then, the corresponding percentages were calculated using the intensity decrease compared with the fluorescence intensity measured without scavengers, and listed in Table 1. It can be seen that the nature of ROS is essentially ^1^O_2_ rather than •O_2_^−^, which is similar to the results of zinc oxide nanoparticles [18]. Since the highly reactive oxidative specie ^1^O_2_ played a significant role in the generated ROS, the samples are supposed to have great photodynamic efficiency.

### 2.3. Photokilling Effects of Samples on HeLa Cells

The photokilling effects of samples on human cervical carcinoma cells (HeLa) were measured under different irradiation. The HeLa cells were first incubated with a medium containing 5.5–21.9 μg·mL^−1^ N-TiO_2_-Pc/TiO_2_-Pc (containing 0.48–1.9 μg·mL^−1^ Pc) for 1 h in the dark. For comparison, cells incubated with the same amount of 0.48–1.9 μg·mL^−1^ Pc were incubated as well. The irradiation time was the same for 420–800 nm and 420–575 nm, hence the contribution of 420–575 nm could be estimated with the same irradiation conditions except the wavelength range.

**Figure 3:**
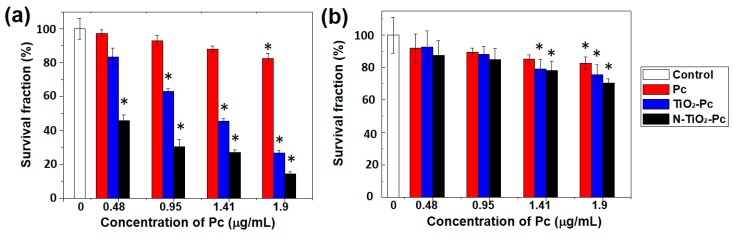
The photokilling effect on HeLa cells treated with 5.5–21.9 μg·mL^−1^ N-TiO_2_-Pc or TiO_2_-Pc with (**a**) 420–800 nm; (**b**) 420–575 nm light irradiation. For comparison, cells incubated with the same amount of 0.48–1.9 μg·mL^−1^ Pc were studied as well. * represents significant difference from the control group (*p* < 0.05).

Under the irradiation of 420–800 nm (15.9 J⋅cm^−2^), the surviving fractions of cells were decreased with the increased concentration of samples, as shown in Figure 3a. Pc showed weak photokilling effect with survival fractions of >83% for all the concentrations. TiO_2_-Pc exhibited higher photokilling effect with the cell survival fractions in the range of 27–83%. N-TiO_2_-Pc showed the highest photokilling effect. The cell survival fraction was below 46% when treated with 5.5 μg·mL^−1^ N-TiO_2_-Pc, and the cell viability was as low as 14% when incubated with 21.9 μg·mL^−1^ N-TiO_2_-Pc.

Under the irradiation of 420–575 nm (7.5 J⋅cm^−2^), Pc did not show great photokilling effect. However, the cell viability dropped to 70% and 78% when treated with 21.9 μg·mL^−1^ N-TiO_2_-Pc and TiO_2_-Pc, respectively (Figure 3b). This indicated that both N-TiO_2_-Pc and TiO_2_-Pc can be activated by 420–575 nm irradiation, while nitrogen-doping can enhance the photodynamic activity of N-TiO_2_-Pc.

## 3. Discussion

From Figure 2a,d, it can be seen that the total ROS production irradiated by 420–575 nm light was about half that irradiated by 420–800 nm light. This was because the power density of the lamp in the range of 420–575 nm was about half of that in the range of 420–800 nm. If the different irradiation light dose of 420–575 nm was same as 420–800 nm, the cell viability was expected to be 36% with 21.9 μg·mL^−1^ N-TiO_2_-Pc. There was a gap of about 22% cell viability for N-TiO_2_-Pc under 420–575 nm light compared with 420–800 nm. This could be explained by the notion that the photokilling effect was determined not only by the total ROS production, but also by the ROS type. Various species contribute differently depending on their lifetimes and diffusion lengths. The natures of ROS were different between 420–575 nm and 575–800 nm. On the other hand, specific ROS were studied in aqueous solutions, as shown in Figure 2 and Table 1, but the ROS would not be the same in the culture medium. In this case, the photokilling effect under 420–575 nm light was not significant (Figure 3b).

**Figure 4:**
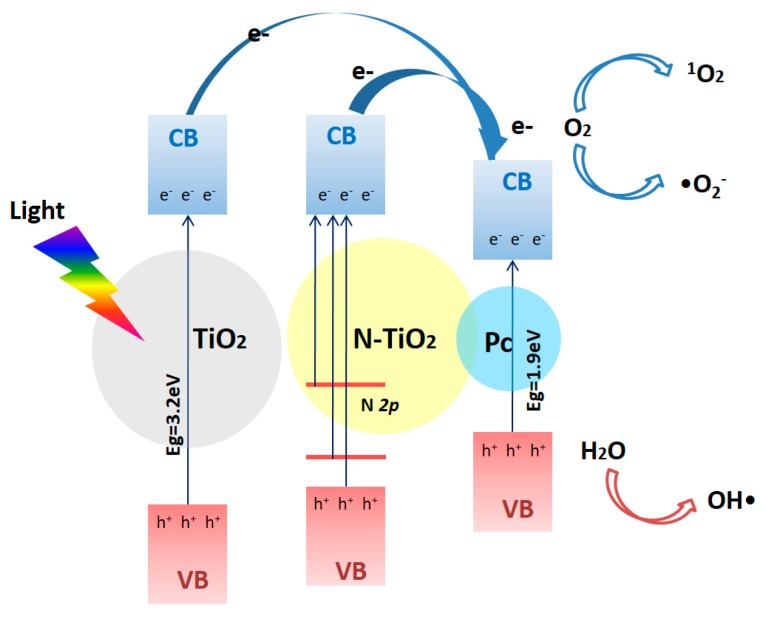
Schematic illustration of a proposed mechanism of ROS production by N-TiO_2_-Pc (or TiO_2_-Pc) under irradiation.

Figure 4 demonstrates a proposed mechanism of ROS production by N-TiO_2_-Pc (or TiO_2_-Pc) under light irradiation. The phthalocyanines in the solid state behave as *p*-type semiconductors with the energy of the band gap at about 1.9 eV [19], which can be excited by red light and mainly generate ^1^O_2_ through energy transfer. Meanwhile, the bandgap of TiO_2_ was narrowed and isolated states of N *2p* were located in the bandgap of N-TiO_2_ due to the nitrogen-doping. As suggested in the theoretical study [20], doping with a 1–2% N concentration could result in a bandgap narrowing of 0.11–0.13 eV, and some N *2p* isolated states lying at 0.25–1.05 eV above the valence-band maximum of TiO_2_. Therefore, the visible light of λ ≤ 575 nm can excite the N-TiO_2_ nanoparticles effectively, and it is more prone to transfer energy from N-TiO_2_ to Pc compared with non-doped TiO_2_. It has been shown through extensive studies that higher N doping amounts narrow the bandgap of TiO_2_ and enhance the visible light absorption. The ROS generation is determined by both the light absorption ability and the quantum efficiency. Since the doped N atoms can serve as electron traps to inhibit the recombination of electrons and holes, the quantum efficiency of photoactivity could be promoted. The photogenerated electrons in the conduction band (CB) can react with oxygen molecules to generate •O_2_^−^ and ^1^O_2_. The production of •O_2_^−^ and ^1^O_2_ by N-TiO_2_-Pc is significantly promoted by nitrogen-doping. On the other hand, the photogenerated positive holes in the valence band (VB) can oxidize water molecules to generate OH•. The results of Reeves proved OH• formation at nanoparticulate TiO_2_ by electron spin resonance (ESR) studies [9]. This was also substantiated by experiments showing that TiO_2_ generated more OH• than N-TiO_2_ [10], which may be attributed to the low mobility of the photogenerated holes trapped in N *2p* levels of N-TiO_2_ [21]. Since OH• contributes less to the photodynamic activity due to its shorter lifetime and lower diffusion length in comparison to •O_2_^−^ [22], it can be understood that TiO_2_-Pc exhibits less photodynamic activity than N-TiO_2_-Pc. Therefore, the results suggest that N-TiO_2_-Pc can be an excellent candidate for a photosensitizer in PDT with wide-spectrum visible irradiation.

## 4. Materials and Methods

### 4.1. Preparation and Characterization of Samples

The chemical agents used in the preparation of N-TiO_2_-Pc or TiO_2_-Pc were anatase TiO_2_ nanoparticles (<15 nm, Sigma-Aldrich Inc., St. Louis, MO, USA), gaseous ammonia (99.999%, Pujiang Inc., Jinhua, China), APTES (3-aminopropyl triethoxysilane, 99%; Aladdin Inc., Astoria, NY, USA), ammonia solution (25%-28%, Tongsheng Inc., Jiangsu, China), methanol (99.5%, Lingfeng Inc., Shanghai, China), and Pc (aluminum phthalocyanine chloride tetrasulfonate; Frontier Scientific Inc., Logan, UT, USA). Other chemical agents included 2-(9H-fluoren-9-ylmethoxycarbonylamino) oxyacetic acid (Fmoc-Aoa, Chem-Impex International, Inc., Bensenville, IL, USA), dimethylformamide (DMF, 98%, Sigma-Aldrich Inc., St. Louis, MO, USA), *N*,*N*-diisopropylethylamine (DIPEA, 99.5%, Sigma-Aldrich Inc., St. Louis, MO, USA), (benzotriazole-1-yloxy) tripyrrolidinophosphonium hexafluorophosphate (PyBOP, 98%, EMD Chemicals, Inc., Gibbstown, NJ, USA), and piperidine (≥99.5%, Sigma-Aldrich Inc., St. Louis, MO, USA).

N-TiO_2_-Pc nanoparticles were synthesized as described in our previous works [11]. Briefly, nitrogen-doped titanium dioxides (N-TiO_2_) were obtained through the calcination of anatase TiO_2_ in an ammonia atmosphere, and the N-dopant concentration was estimated to be 1.3%, as reported in our previous study [15,23]. Then, the NPs were modified with the amino silanization method [14] and coupled with Pc [11]. TiO_2_-Pc nanoparticles were synthesized following the same procedure except for the calcination of anatase TiO_2_ in an ammonia atmosphere. As previously reported [11], every 21.9 μg N-TiO_2_-Pc or TiO_2_-Pc contains 1.9 μg Pc, and the nanoparticles can be stably dispersed in aqueous solution.

The ultraviolet-visible (UV/Vis) absorption spectra of the N-TiO_2_-Pc, TiO_2_-Pc, and Pc samples were measured with a UV/Vis spectrometer (Shimadzu, UV3101pc, Tokyo, Japan).

### 4.2. Measurement of Reactive Oxygen Species (ROS)

The photo-induced generations of ROS in N-TiO_2_-Pc, TiO_2_-Pc, and Pc solutions were measured via 2′7′-dichlorofluorescein (DCFH). With light irradiation, the non-fluorescent DCFH reacts quickly with photo-induced ROS to form fluorescent DCF (2′7′-dichlorofluorescein). Thus, by measuring the fluorescence intensity of DCF, the relative yield of the produced ROS could be estimated. The DCFH solutions were prepared from the diacetate form DCFH (DCFH-DA) (Sigma-Aldrich Inc., St. Louis, MO, USA) by adding 0.5 mL of 1 mM DCFH-DA in methanol into 2 mL of 0.01 M NaOH. The mixture was kept in the dark for 30 min at room temperature before it was neutralized with 10 mL sodium phosphate buffer (pH = 7.2) [10,24]. Then, samples in phosphate buffered saline (PBS) solutions were individually mixed with DCFH (25 µM) before irradiation.

To evaluate the generations of specific reactive species, an OH•-sensitive fluorescence probe, 2-[6-(4-aminophenoxy)-3-oxo-3H-xanthen-9-yl]-benzoic acid (APF, Cayman Chemical, Ann Arbor, MI, USA) [25], and an •O_2_^−^/H_2_O_2_ sensitive fluorescence probe, dihydrorhodamine 123 (DHR, Sigma-Aldrich Inc., St. Louis, MO, USA) [26], were used. Samples were mixed with APF (50 µM) or DHR (125 µM) before irradiation. When photo-induced OH• or •O_2_^−^/H_2_O_2_ reacts with the non-fluorescent APF or DHR, the two probes can be converted to fluorescents quickly.

The sample solutions mixed with probes (or quenchers) were irradiated by a 150-W tungsten halogen lamp with different light filters for 5 min, respectively. The light of 420–800 nm was obtained with a 420 nm-longpass filter and an 800 nm-shortpass filter. The light of 420–575 nm was obtained with a 420 nm-longpass filter and a 575 nm-shortpass filter. The irradiation power densities were 17.8 mW⋅cm^−2^ (420–800 nm) and 8.4 mW⋅cm^−2^ (420–575 nm). At the same time, the fluorescence spectra were recorded by a fluorescence photometer (Hitachi, F-2500, Tokyo, Japan) with an interval of 1 min, and the fluorescent intensities were compared. The fluorescence intensities increased with the irradiation time, and the lines in Figure 2 were fitted linearly.

To further study the proportion of different specific ROS generated by the samples with light irradiation, several quenchers for specific ROS were used, including superoxide dismutase (SOD, 3IU, Beyotime, Jiangsu, China) for •O_2_^−^ [17] and glycerol (99%, Sangon Biotech, Shanghai, China) for ^1^O_2_/•O_2_^−^ [16]. First, the fluorescence intensities of DCF with samples under irradiation were recorded as references, and the intensity versus time was a linear line with a slope noted as *S_REF_*. Then, specific quenchers were respectively added into the DCFH and sample solutions before irradiation, where the concentrations of DCFH and samples were the same as that of the references. During irradiation, the fluorescence intensities of DCF in the presence of specific ROS quenchers were also recorded with an interval of 1 min. The lines of intensity with quenchers versus time were linear as well, and the slopes were noted as *S_Q_*. Hence, the specific ROS percentages were obtained as $1-\frac{S_{Q}}{S_{REF}}$, and are listed in Table 1.

### 4.3. Cell Culture and Cytotoxicity Assay

HeLa cells were seeded in 96-well plates containing Dulbecco’s modified Eagle’s medium (DMEM) (Gibco, Waltham, MA, USA) with 10% (*v*/*v*) fetal bovine serum (Sijiqing Inc., Hangzhou, China), and incubated in a fully humidified incubator at 37 °C with 5% CO_2_ until reaching 80% confluence. Cells were incubated with a medium containing 5–20 μg·mL^−1^ N-TiO_2_-Pc or TiO_2_-Pc (containing 0.48–1.9 μg·mL^−1^ Pc) for 1 h in the dark. For comparison, cells incubated with 0.48–1.9 μg·mL^−1^ Pc were studied as well. Then, cells were incubated in fresh medium after washing three times and irradiated by the 150-W tungsten halogen lamp with different light filters, respectively. The irradiation time was same for 420–800 nm and 420–575 nm, therefore the visible-light illumination doses for cells were 15.9 J⋅cm^−2^ with 420–800 nm, and 7.5 J⋅cm^−2^ with 420–575 nm. The cells were incubated in the dark for 24 h before the cell viability study.

The cell viability assays were conducted by a modified 3-(4,5-dimethyl-2-thiazolyl)-2,5-diphenyl-2-H-tetrazolium bromide (MTT) method using WST-8 (2-(2-methoxy-4-nitrophenyl)-3-(4-nitrophenyl)-5-(2,4-disulfophenyl)-2H tetrazolium, monosodium salt) (Beyotime, Jiangsu, China). To each well, 100 μL culture medium with 10 μL of WST-8 solution was added. The cells were then incubated at 37 °C with 5% CO_2_ for 2 h, and the absorbance of each well at 450 nm was recorded using a microplate reader (Bio-Tek Instruments Inc., Winooski, VT, USA). The absorbance at 450 nm before adding WST-8 was measured, and needed to be deducted to avoid any influence from nanoparticle samples. Cells incubated in DMEM medium without any treatment were used as control groups. Each experiment was conducted and measured independently at least three times.

## Figures and Tables

**Table 1:** Specific ROS percentage (%) of total ROS under different irradiation wavelengths.

	•O_2_^−^		^1^O_2_/•O_2_^−^	
Excitation Range	420–800 nm	420–575 nm	420–800 nm	420–575 nm
N-TiO_2_-Pc	12.6 ± 0.3	20.9 ± 0.7	52.3 ± 1.8	66.6 ± 1.6
TiO_2_-Pc	7.6 ± 0.2	10.0 ± 0.1	69.8 ± 1.7	63.7 ± 0.1
Pc	7.5 ± 0.1	--	65.6 ± 0.9	--
